# Supplementary material for: A network dynamical simulation model for the study of antibiotic resistance in microbial communities
Source: Gut Microbes. 2026 Sep 19;18(1):2734703. doi: 10.1080/19490976.2026.2734703 (PMC13596919; doi:10.1080/19490976.2026.2734703)
Supplement: Supplementary file 1 [file KGMI_A_2734703_SM9053.zip › supplementary_file_1/Figures/ws_delta_clustering_n1000.pdf]

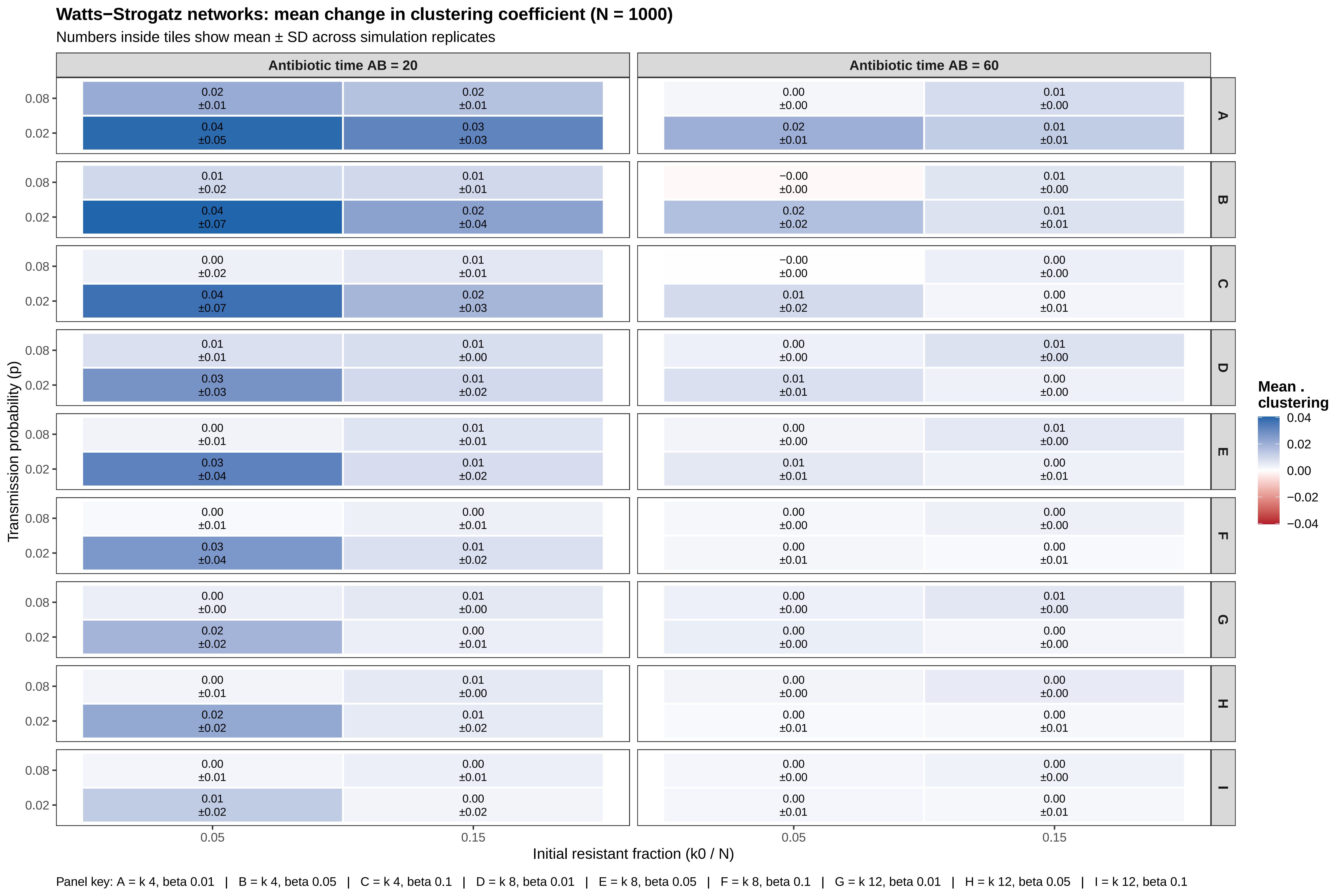
Watts−Strogatz networks: mean change in clustering coefficient (N = 1000)
Numbers inside tiles show mean ± SD across simulation replicates
Antibiotic time AB = 20
Antibiotic time AB = 60
A
B
C
D
E
F
G
H
I
0.08
0.02
Transmission probability (p)
Initial resistant fraction (k0 / N)
0.05
0.15
Mean . clustering
0.04
0.02
0.00
−0.02
−0.04
0.02 ±0.01
0.02 ±0.01
0.04 ±0.05
0.03 ±0.03
0.00 ±0.00
0.01 ±0.00
0.02 ±0.01
0.01 ±0.01
0.01 ±0.02
0.01 ±0.01
0.04 ±0.07
0.02 ±0.04
−0.00 ±0.00
0.01 ±0.00
0.02 ±0.02
0.01 ±0.01
0.00 ±0.02
0.01 ±0.01
0.04 ±0.07
0.02 ±0.03
−0.00 ±0.00
0.00 ±0.00
0.01 ±0.02
0.00 ±0.01
0.01 ±0.01
0.01 ±0.00
0.03 ±0.03
0.01 ±0.02
0.00 ±0.00
0.01 ±0.00
0.01 ±0.01
0.00 ±0.00
0.00 ±0.01
0.01 ±0.01
0.03 ±0.04
0.01 ±0.02
0.00 ±0.00
0.01 ±0.00
0.01 ±0.01
0.00 ±0.01
0.00 ±0.01
0.00 ±0.01
0.03 ±0.04
0.01 ±0.02
0.00 ±0.00
0.00 ±0.00
0.00 ±0.01
0.00 ±0.01
0.00 ±0.00
0.01 ±0.00
0.02 ±0.02
0.00 ±0.01
0.00 ±0.00
0.01 ±0.00
0.00 ±0.00
0.00 ±0.00
0.00 ±0.01
0.01 ±0.00
0.02 ±0.02
0.01 ±0.02
0.00 ±0.00
0.00 ±0.00
0.00 ±0.01
0.00 ±0.01
0.00 ±0.01
0.00 ±0.01
0.01 ±0.02
0.00 ±0.02
0.00 ±0.00
0.00 ±0.00
0.00 ±0.01
0.00 ±0.01
Panel key: A = k 4, beta 0.01 | B = k 4, beta 0.05 | C = k 4, beta 0.1 | D = k 8, beta 0.01 | E = k 8, beta 0.05 | F = k 8, beta 0.1 | G = k 12, beta 0.01 | H = k 12, beta 0.05 | I = k 12, beta 0.1
